# Supplementary material for: A new member of fossil balaenid (Mysticeti, Cetacea) from the early Pliocene of Hokkaido, Japan
Source: R Soc Open Sci. 2020 Apr 22;7(4):192182. doi: 10.1098/rsos.192182 (PMC7211833; doi:10.1098/rsos.192182)
Supplement: Supplementary 3 [file rsos192182supp3.docx]

**List of characters.**

The all characters are cited from Buono et al. (2017).

**Skull**

1. Length of rostral portion of maxilla anterior to antorbital notch: less than bizygomatic width (0); equal to or greater than bizygomatic width (1); more than one and a half times the bizygomatic width (2).

2. Portion of rostrum anterior to nasals in lateral view: below the level of the supraoccipital (0); raised to the level of the supraoccipital (1); raised above the level of the supraoccipital (2)

3. Lateral edge of maxilla in cross section: forms an angle of more than 45 degrees (0); lateral edge is flattened with an angle of less than 45 degrees (1).

4. Lateral border of maxilla anterior to antorbital notch (or homologous point on rostrum) in dorsal view: concave (0); straight or slightly convex (1); broadly convex (2).

5. Transverse width of maxilla at midpoint: distinctly less than that of the premaxilla (0); roughly equal to or up to twice the width of the premaxilla (1); more than twice the width of the premaxilla (2).

6. Premaxilla in dorsal view: widens at anterior end (0); portion anterior to nasal opening narrows or remains the same width anteriorly (1).

7. Premaxilla adjacent to and anterior to narial fossa: elevated above the maxilla and forming a distinct lateral face (0); continuous or nearly continuous with the maxilla (1).

8. Premaxilla adjacent to and at posterior edge of nasal opening: does not clearly overhang maxilla (0); premaxilla overhangs maxilla (1).

9. Anterior portions of premaxillae: firmly contact each other (0); premaxillae are separated or only loosely contact along their entire length (1).

10. Suture between maxilla and premaxilla on rostrum: firmly articulated (0); loose (1).

11. Antorbital process: absent (0); present and defined by a steep face clearly separating the posterolateral corner of the maxilla from its more anterior rostral portion (1); present as a distinct anterior projection lateral to antorbital notch (2).

12. Anterior border of supraorbital process lateral to ascending process of the maxilla: b by lacrimal and maxilla (0); b by lacrimal only (1); as state 0, but with the antorbital process of the maxilla and the anterior border of the supraorbital process separated by a basin (2); as state 0 but with maxilla overriding the anteriormost border of the supraorbital process (3).

13. Distinct pocket between the ascending process of the maxilla dorsally and the supraorbital process ventrally: absent (0); present (1).

14. Lateral process of maxilla (*sensu* Marx, Lambert & Uhen, 2016): absent (0); present and clearly distinct from ascending process of maxilla (1); present and confluent with ascending process of maxilla (2).

15. Lateral process of the maxilla: directed posterolaterally at an oblique angle to the anteroposterior axis of the cranium; directed laterally, nearly perpendicular to the anteroposterior axis of the cranium.

16. Posterior portion of palatal surface of maxilla: flattened or slightly concave (0); medial portion of maxilla forms a longitudinal keel b laterally by a shallow longitudinal trough (1); medial portion forms a keel without any adjacent trough (2).

17. Palatal surface of anterior part of rostrum: flat or gently concave (0); bears pronounced longitudinal keel formed by the vomer and the medial edges of the maxillae (1).

18. Exposure of premaxilla on palate: exposed along at least one third of the medial border of the maxilla (0); limited in extent to less than one third of the medial border of the maxilla (1).

19. Palatal window exposing vomer: absent (0); present (1); narrow and variable exposure of vomer along most or all of the midline of the rostrum (2); vomer broadly exposed along the midline of the rostrum (3).

20. Palatal nutrient foramina and sulci: absent (0); present (1).

21. Outline of suture between maxillae, vomer and palatines: roughly straight transversely or bowed anteriorly (0); forms a posteriorly pointing V shape (1); anterior margins of palatines form two separate and posteriorly pointing U shapes (2).

22. Anteriormost point of palatine: located in line with or posterior to the level of the antorbital notch or equivalent point on rostrum (0); located anterior to the level of the antorbital notch (1).

23. Anterior portion of palatine distinctly concave transversely and forming a sharp median crest: absent (0); present (1).

24. Anterior edge of nasal fossa: located in posterior three quarters of rostrum (0); located in anterior quarter of rostrum (1).

25. Facial portion of rostrum in lateral view: step-like (0); straight (1); concave (2).

26. Rostrum shape: width at antorbital notches or equivalent point on rostrum less than 80% the length of the rostrum, as measured from its tip to the antorbital notches (0); width at antorbital notches or equivalent point more than 80% the length of the rostrum (1).

27. Teeth in adult individuals: present (0); absent or vestigial (1).

28. Skull length about one third or more of total body length: absent (0); present (1).

29. Cranial asymmetry: present (0); absent (1).

30. Diameter of orbit as measured between the distalmost points of the preorbital and postorbital processes: less than 25% of bizygomatic width (0); 25% or more (1).

31. Anterior edge of supraorbital process lateral to ascending process of maxilla with the skull in dorsal view: oriented transversely or pointing anteriorly (0); pointing posteriorly (1); linguiform and tapering to a point (2).

32. Outline of anterior edge of supraorbital process in dorsal view: roughly straight or concave (0); distinctly sinusoidal (1).

33. Transverse width of anterior edge of supraorbital process lateral to ascending process of maxilla: longer than or equal to the combined transverse width of the adjacent rostral bones, as measured from the sagittal plane to the lateral border of the ascending process of the maxilla (0); shorter than the combined transverse width of the adjacent rostral bones (1).

34. Posterior border of supraorbital process in dorsal view: concave (0); straight (1).

35. Supraorbital process of frontal in anterior view: horizontal or nearly horizontal (0); gradually slopes away lateroventrally from the skull vertex (1); as state 1 but with the lateral portion of the supraorbital being nearly horizontal, thus causing the latter to appear concave in anterior view (2); abruptly depressed to a level noticeably below the vertex, with the lateral skull wall above the supraorbital formed by both parietal and frontal (3).

36. Anterior and posterior borders of supraorbital process in dorsal view: roughly parallel or converging medially (0); converging laterally (1).

37. Width of supraorbital process as measured in a straight line from the lateralmost point of the postorbital process to the intertemporal constriction: equal to or shorter than the anteroposterior length of the supraorbital process above the orbit (0); up to twice the length above the orbit (1); more than twice the length above the orbit (2).

38. Postorbital process in dorsal view: oriented posteriorly (0); oriented laterally (1); oriented posterolaterally (2); short and not markedly projecting in any direction (3).

39. Postorbital process in lateral view: pointed or rounded (0); forms and anteroposteriorly elongate triangle with a flattened posterior face (1).

40. Orbital rim of supraorbital process of frontal in lateral view: dorsoventrally thin (0); thickened with a flat lateral surface (1); thickened with a rounded lateral surface (2).

41. Position of anteriormost point of supraorbital process in dorsal view: in line with the posterior extremity of the nasals or passing through the nasals (0); at the same level as the anterior extremity of the nasals (1); anterior to the anterior extremity of the nasals (2).

42. Position of the dorsal margin of the orbit (in lateral view, with skull resting on a horizontal surface): above or in line with the vertex of the skull (0); located roughly a half of distance between the vertex of the skull and the ventral surface of the postglenoid process (1) located a 2/3 the vertical distance between the vertex of the skull and the ventral surface of the postglenoid process (2); located a 1/3 the vertical distance between the vertex of the skull and the ventral surface of the postglenoid process (3)

43. Lacrimal in dorsal view: situated entirely lateral to the ascending process of the maxilla (0); lacrimal extends medially and separates the lateral corner of the ascending process from the more anterior portion of the maxilla (1).

44. Contact of jugal with zygomatic process of squamosal: the two bones overlap dorsoventrally (0); little or no overlap (1).

45. Anteriormost portion of jugal broadly underlapped by maxilla: absent (0); present (1).

46. Optic canal in ventral view: ventrally open (0); medial portion is enclosed by anterior and/or posterior bony laminae (1).

47. Postorbital ridge along medial portion of optic canal: absent or anteroposteriorly thin, with the optic canal running adjacent to the posterior border of the supraorbital process (0); well developed and thickened, thus resulting in the displacement of the optic canal away from the posterior border of the supraorbital process (1).

48. Maxillary infraorbital plate (*sensu* Marx, Lambert & Uhen, 2016): absent (0); present (1).

49. Maxillary window (*sensu* Marx, Lambert & Uhen, 2016) originating from posterior border of infraorbital plate: absent (0); present (1).

50. Anteromedial corner of supraorbital process extending to a point medial to antorbital notch: absent (0); present (1).

51. Preorbital region of frontal in lateral view: thickened compared to more central portions of the orbit (0); dorsoventrally flat (1).

52. Enlarged dorsal infraorbital foramen on ascending process of maxilla, opening into a posterodorsally directed sulcus: absent (0); present (1).

53. Premaxillary sac fossa: absent (0); present (1).

54. Premaxillary foramen: absent (0); present (1).

55. Suture between maxilla and frontal: contact between the bones is straight or maxilla overrides anteromedial corner of the frontal (0); maxilla overrides half or more of the frontal (1).

56. Lateral borders of ascending process of maxilla: lateral edges convergent with the process tapering to a point (0); lateral edges parallel or divergent posteriorly (1).

57. Triangular wedge of frontal separating ascending process of maxilla from nasal orpremaxilla: absent (0); present (1).

58. Posterior ends of ascending processes of maxillae in dorsal view: separated by either frontals or both nasals and premaxillae (0); converging towards the midline and separated by nasals only (1); contact each other medially (2).

59. Relative position of posteriormost edge of ascending process of maxilla in dorsal view: approximately in transverse line with, or posterior to, posterior edge of nasal (0); anterior to posterior edge of nasal (1).

60. Shape of posterior border of ascending process of maxilla: pointed or rounded (0); squared off (1).

61. Lateral profile of cranium along exposure of parietals on vertex: dorsal edge of parietal ascends steeply towards posterior edge of skull at an angle of 10 degrees or more, measured relative to the lateral edge of rostrum (0); dorsal edge of parietal is low to flat with an angle of less than 10 degrees (1).

62. Length of nasal relative to bizygomatic width: less than 50% of bizygomatic width (0); more than 50% of bizygomatic width (1).

63. Lateral margins of nasal: parallel (0); posteriorly convergent (1).

64. Anterior margins of nasals: roughly straight or U-shaped (0); form a distinct, posteriorly pointing W-shape (1); with point on midline and a gap on each side between premaxilla and nasal (2);

65. Dorsal surface of nasals: flattened (0); developed into a sagittal keel (1).

66. Separation of posterior portions of nasals along sagittal plane by narial process of frontal: present (0); absent (1).

67. Zygomatic process of squamosal and exoccipital in dorsal view: clearly separated by an angle (0); posterior border of zygomatic process and lateral edge of exoccipital are confluent, forming a continuous or nearly continuous lateral skull border (1).

68. Orbitotemporal crest: positioned along posterior border of supraorbital process, with the origin of the temporal muscle facing posteriorly or posteroventrally (0); absent or positioned on the dorsal surface of the supraorbital process, with the origin of the temporal muscle facing posterodorsally or dorsally (1).

69. Area enclosed by orbitotemporal crest on supraorbital process of frontal: forms less than half of the dorsal surface of the supraorbital process (0); covers half or more of the dorsal surface of the supraorbital process (1).

70. Outline and orientation of orbitotemporal crest: subparallel to posterior border of supraorbital process (0); distal half oriented distinctly posterolaterally and approaching the posterolateral corner of the supraorbital process (1); as state 1, but with the crest terminating halfway along the posterior border of the supraorbital process (2); as state 2, but with the crest being distinctly U-shaped (3).

71. Shape of temporal fossa: longer anteroposteriorly than wide transversely, or as wide as long (0); wider than long (1).

72. Intertemporal constriction: longer anteroposteriorly than wide transversely (0); as state 1, but with the temporal fossa forming a large parasagittal oval (1); wider transversely than long anteroposteriorly (2).

73. Exposure of frontal on skull vertex: broadly exposed (0); anteroposteriorly compressed or absent (1).

74. Parietal and interparietal: anteriormost point located no further forward than postorbital process (0); anteriormost point in line with supraorbital process (1).

75. Outline of fronto-parietal suture: straight or lobate (0); frontals projects posteriorly along the sagittal plane and separate the left and right parietal anteriorly (1); highly irregular (2).

76. Parietal in lateral view: as long or longer anteroposteriorly than high dorsoventrally (0); higher dorsoventrally than long anteroposteriorly (1).

77. Spreading of anterolateral portion of parietal on to posteromedial corner of supraorbital process of frontal: absent (0); present (1).

78. Anteriormost point of parietal and interparietal: more posterior than the posterior border of the ascending process of the maxilla (0); more anterior than or in line with the posterior border of the ascending process of the maxilla (1).

79. Anteriormost point of supraoccipital in dorsal view: located posterior to or in line with the anterior border of the squamosal fossa (0); in line with temporal fossa, but posterior to the apex of the zygomatic process of the squamosal (1); in line with or located anterior to the level of the apex of the zygomatic process of the squamosal (2); in line with the anterior half or anterior edge of the supraorbital process (3).

80. Anteroposterior position of posterior apex of nuchal crest: posterior to the occipital condyle (0); anterior to or in line with the posteriormost point of the occipital condyle (1).

81. Mediolateral position of posterior apex of nuchal crest: aligned with the medial half or halfway point of the temporal fossa (0); approaching the level of the lateral border of the temporal fossa (1).

82. Distinct tubercle at junction of parieto-squamosal suture and supraoccipital: absent (0); present (1).

83. Exposure of alisphenoid within or at ventral border of temporal fossa: exposed on temporal wall of skull and contributing to orbital fissure (0); alisphenoid separated from orbital fissure or not exposed on temporal skull wall (1).

84. Postparietal foramen located at junction of parietal and squamosal: absent (0); present (1).

85. Zygomatic process of squamosal dorsoventrally expanded in lateral view: absent (0); present, with the zygomatic process being distinctly higher dorsoventrally than wide transversely (1).

86. Orientation of zygomatic process of squamosal in dorsal view: directed anteromedially (0); directed anteriorly or slightly anterolaterally (1); directed anterolaterally (2).

87. Zygomatic process of squamosal in lateral or ventral view: tapering anteriorly (0);expanded anteriorly, thus forming a central constriction (1).

88. Twisting of zygomatic process of squamosal: absent (0); present, with the zygomatic process being partially twisted clockwise on the left and anticlockwise on the right (1); as state 1, but with the process twisted almost 90 degrees (2).

89. Position of apex of zygomatic process of squamosal: situated entirely posterior to the postorbital process (0); closely apposed to the postorbital process, or situated ventral to the latter (1).

90. Apex of zygomatic process of squamosal deflected anteroventrally: absent (0); present (1).

91. Supramastoid crest of zygomatic process of squamosal (skull in lateral view): present (0); absent (1).

92. Size of squamosal including zygomatic and postglenoid processes: longer anteroposteriorly than high dorsoventrally, or about as high as long (0); distinctly higher than long (1).

93. Parieto-squamosal suture shaped like a crest or ridge: absent or low (0); present and distinctly elevated (1).

94. Squamosal prominence (*sensu* Fordyce & Marx, 2013): present as a projection on the crest delimiting the lateral or posterolateral edge of the squamosal fossa (0); absent (1).

95. Transverse width of squamosal lateral to exoccipital: width equal to or greater than 15% of the distance between the sagittal plane and the lateral edge of the exoccipital (0); exposed portion of squamosal is less than 15% of that distance (1).

96. Length of squamosal fossa relative to maximum transverse width of temporal fossa, as measured in a straight line from the posteriormost point of the temporal fossa to the posteriormost point of the nuchal crest: length of squamosal fossa is three quarters the width of the temporal fossa or longer (0); length of squamosal fossa is less than three quarters the width of the temporal fossa (1).

97. Squamosal cleft (*sensu* Marx, Lambert & Uhen, 2016): absent (0); present (1).

98. Squamosal crease: absent (0); present (1).

99. Paired tubercles on supraoccipital: absent (0); limited to low ridges forming the lateral edges of a medial fossa (1); present (2).

100. Lateral edge of supraoccipital in dorsal view: convex (0); straight (1); concave (2); sigmoidal (3).

101. Anterior border of supraoccipital shield: rounded or pointed (0); squared (1).

102. Overall outline of supraoccipital in dorsal view: rounded (0); triangular (1).

103. Anterior half of dorsal surface of supraoccipital: concave (0); flat or convex (1).

104. External occipital crest: absent or faint (0); restricted to anterior half of supraoccipital shield (1); present and running all the way along the supraoccipital shield (2).

105. Tip of postglenoid process in lateral view: curving anteriorly (0); pointing ventrally (1); pointing posteriorly (2).

106. Ventral edge of postglenoid process in lateral view: approximately in line with or dorsal to the ventral edge of the exoccipital (0); extending well ventral to the ventral edge of the exoccipital (1).

107. Orientation of postglenoid process in posterior view: ventrolateral (0); ventral (1); ventromedial (2).

108. Outline of postglenoid process in anterior or posterior view: parabolic (0); as state 0, but with lateral and medial edges parallel or concave (1); as state 1, but distinctly wider transversely than high dorsoventrally (2); triangular (3); trapezoidal, with a ventrally directed medial border (4).

109. Twisting of postglenoid process in ventral view: absent (0); twisted clockwise on the left side and anticlockwise on the right side so that the glenoid cavity faces anteromedially (1).

110. Position of base of postglenoid process in ventral or posterior view: in line with the lateral edge of the skull (0); shifted away medially from the lateral edge of the skull (1).

111. Medial border of postglenoid process in ventral view: confluent with more medial portion of squamosal (0); offset from remainder of squamosal by a distinct ridge (1).

112. Choanal margin of palatine in ventral view: absent (0); straight or convex (1); concave (2); forms a longitudinal notch (3).

113. Pterygoid in ventral view: partially or entirely exposed (0); palatine almost completely covers pterygoid and extends on to the hamular process (1).

114. Anteriormost point of pterygoid sinus fossa: located anterior to foramen pseudovale (0); approximately in line with anterior edge of foramen pseudovale (1); located posterior to anterior edge of foramen pseudovale (2).

115. Dorsal lamina of pterygoid: absent or restricted to anteromedial quarter of pterygoid sinus fossa (0); present and covering half or more of ventral exposure of alisphenoid within pterygoid sinus fossa (1).

116. Shape of pterygoid hamulus: finger-like (0); expanded into a dorsoventrally flattened plate flooring the pterygoid sinus fossa (1); triangular and wing-like (2); reduced in size or almost absent (3).

117. Position of pterygoid hamulus in ventral view: located directly adjacent to the sagittal plane and almost contacting each other (0); well separated from each other (1).

118. Position of foramen pseudovale: foramen located within squamosal or between squamosal and pterygoid, and opening anterolaterally or laterally (0); as state 0, but with foramen opening posteriorly (1); foramen lies within pterygoid (2).

119. Foramen pseudovale raised above more lateral portions of squamosal in ventral view: absent (0); present (1).

120. Fossa on squamosal for reception of sigmoid process of tympanic bulla: present (0); absent or poorly defined (1).

121. Base of postglenoid process in ventral view: in transverse line with or located posterior to the posterior half of the tympanic bulla (0); in transverse line with or located anterior to the anterior half of the tympanic bulla (1); in transverse line with the anteroposterior centre of the tympanic bulla (2).

122. Ventral border of sagittal part of vomer (nasals septum) in ventral view: posteriormost portion projects beyond the posterior border of the palatines and is visible in ventral view (0); completely covered by palatines (1).

123. Basioccipital crest: narrow transversely (0); wide and bulbous (1).

124. Lateral border of basioccipital crest in ventral view: straight (0); concave (1).

125. Orientation of basioccipital crests in ventral view: diverging posteriorly (0); parallel or subparallel (1).

126. Ventromedial corner of paroccipital process in posterior view: located more ventrally than the basioccipital crest (0); level with or more dorsal than the basioccipital crest (1).

127. Posteriormost point of exoccipital in dorsal view: located more anteriorly than posterior edge of occipital condyle (0); level with or posterior to posterior edge of condyle (1).

**Hyoid**

128. Outline of stylohyal in cross section: cylindrical (0); flattened (1).

129. Orientation of thyrohyal in dorsal or ventral view: oriented posteriorly (0); oriented laterally (1).

130. Shape of thyrohyal: cylindrical (0); flattened and wing-like (1); plate-like (2).

131. Ankylosed basihyal and thyrohyals: absent (0); present (1).

**Periotic**

132. Dorsal and medial elongation of pars cochlearis towards cranial cavity: absent (0); present (1); as state 1, but with only the anterior side of the pars cochlearis being elongated (2).

133. Attachment of anterior process to pars cochlearis: absent (0); present (1).

134. Anterior process of periotic in lateral view: squared off or rounded (0); triangular (1); anterior border of process is two-bladed and L-shaped (2).

135. Shape of anteroventral angle of anterior process of periotic in medial or lateral view: rounded or forms a relatively blunt angle (0); slender and tapering to a point (1).

136. Ventral edge of anterior process of periotic in medial view: at the same level or dorsal to ventral edge of pars cochlearis (0); ventral to ventral profile of pars cochlearis (1).

137. Dorsal deflection of anterodorsal corner of anterior process: absent (0); present (1).

138. Anterior process transversely compressed and blade-like: absent (0); present (1).

139. Length of anterior process of periotic: shorter than the anteroposterior length of the pars cochlearis, as measured from the anterior border of the pars cochlearis to the medial border of the fenestra rotunda (0); same length or longer than the pars cochlearis (1).

140. Anteroexternal sulcus: forms an oblique or vertical groove on lateral side of anterior process, immediately anterior to lateral tuberosity (0); absent (1).

141. Pyramidal process: absent (0); present (1).

142. Articulation of anterior process of periotic and tympanic bulla: no contact, or contact with accessory ossicle via fovea epitubaria on the anterior process of the periotic (0); accessory ossicle or homologous region on periotic fused to bulla (1).

143. Anterior bullar facet: present (0); absent (1).

144. Lateral tuberosity of anterior process: absent or relatively small and rounded (0); well developed and distinctly triangular (1); hypertrophied and blade-like (2); forms a distinct shelf (3).

145. Position of lateral tuberosity: situated posterolateral to anterior pedicle of tympanic bulla or fovea epitubaria (0); situated lateral or anterolateral to anterior pedicle of tympanic bulla or fovea epitubaria (1).

146. Body of periotic lateral to pars cochlearis hypertrophied: absent (0); present laterally and ventrally (1); present laterally only (2).

147. Mallear fossa: well excavated and possessing a clearly defined rim (0); present only as a depression with diffuse edges (1).

148. Distinct ridge delimiting insertion surface of tensor tympani on medial side of anterior process: absent (0); absent, but insertion surface distinctly excavated (1); present (2).

149. Dorsal extension of attachment area for tensor tympani on medial side of anterior process: absent or indistinct (0); present as a deeply excavated canal (1).

150. Anteromedial corner of pars cochlearis in ventral view: developed as a rounded, anteroposterior ridge (0); angular and projecting medially, resulting in a flattened ventral surface of the pars cochlearis (1); smooth and rounded (2).

151. Promontorial groove on medial side of pars cochlearis: present, but relatively shallow (0); present and deeply excavated (1); present and forming a distinct constriction, separating a smooth and rounded ventral portion of the pars cochlearis from a flattened and striated dorsal one (2); absent (3).

152. Caudal tympanic process in posteromedial view: well separated from crista parotica (=facial crest) (0); narrow separation or contact (1).

153. Posteromedial corner of pars cochlearis medial to fenestra rotunda: rounded and level with fenestra rotunda (0); inflated and projecting posteriorly beyond fenestra rotunda (1).

154. Morphology of caudal tympanic process: developed as a posteriorly extending triangular shelf (0); as state 0, but with the ventral border bulging ventrally (1); as state 0 but pointing posterodorsally (2); developed as a robust, ventrally directed projection (3); absent or poorly developed (4).

155. Anteroposterior alignment of proximal opening of facial canal, internal acoustic meatus and aperture for cochlear aqueduct: present (0); absent (1).

156. Anteroposterior alignment of aperture for cochlear aqueduct and aperture for vestibular aqueduct: absent (0); present (1).

157. Prominent septum dividing foramina for vestibular and cochlear nerves within internal acoustic meatus: present (0); absent (1).

158. Shape of aperture for cochlear aqueduct: round with sharply defined dorsal margins (0); slit-like (1).

159. Size of aperture for cochlear aqueduct: smaller than aperture for vestibular aqueduct (0); approximately the same size (1).

160. Aperture for cochlear aqueduct and fenestra rotunda: separate (0); confluent (1).

161. Superior process: present as a distinct crest forming the lateral wall of the suprameatal fossa (0); the lateral border of the suprameatal fossa is low and not clearly defined (1).

162. Suprameatal fossa hypertrophied: absent (0); present (1).

163. Development of crista transversa: depressed well below the rim of the internal acoustic meatus (0); well developed and reaching the cerebral surface of the pars cochlearis (1).

164. Hiatus Fallopii: absent or small opening located anterior or anteroventral to proximal opening of facial canal (0); as state 0, but with the hiatus Fallopii being well developed and large (1); anterior border of proximal opening of facial canal is continuous with the hiatus Fallopii and shaped like a fissure (2).

165. Size of proximal opening of facial canal: no more than half the size of the internal acoustic meatus (0); more than half the size of the internal acoustic meatus (1).

166. Squamosal flange located posterior to lateral tuberosity: absent (0); present (1).

167. Articulation surfaces on posterior processes of tympanic bulla and periotic: unfused (0); fused in adults to form compound posterior process (1).

168. Morphology of facial sulcus on distal half of compound posterior process: absent or relatively shallow sulcus with equally defined anterior and posterior borders (0); marked groove with an elevated anterior border (1); deeply incised canal (2); as state 2, but with the facial sulcus being partially or entirely floored, tubular and present along the entire ventral surface of the compound posterior process (3).

169. Position of facial sulcus on compound posterior process in ventral view: facial sulcus runs close to or along the posterior border of the compound posterior process (0); facial sulcus located centrally on the ventral surface of the compound posterior process (1).

170. Orientation of compound posterior process in ventral view, with periotic being in situ: oriented posterolaterally with respect to the longitudinal axis of the anterior process of the periotic (0); oriented at a right angle to the axis of the anterior process (1).

171. Shape of compound posterior process: cylindrical or slightly conical (0); short and stocky (1); flattened anteroposteriorly (2); forms a distinct plug (3).

172. Exposure of compound posterior process on lateral skull wall: external surface of compound posterior process is absent or poorly defined (0); external surface is present but distinct from lateral skull wall (1); lateral surface is expanded and firmly integrated into the posterior process being concave and defined by a distinct ridge separating it from the ventral surface (3).

173. Neck of compound posterior process markedly constricted: absent (0); present (1).

**Tympanic bulla**

174. Anterior border of bulla in dorsal or ventral view: obliquely truncated (0); squared (1); rounded (2); pointed (3).

175. Anterior portion of bulla transversely wider than posterior portion in ventral view: absent (0); present (1).

176. In situ orientation of main axes of tympanic bullae in ventral view: diverging posteriorly (0); parallel (1); diverging anteriorly (2).

177. Position of dorsal origin of lateral furrow: located along posterior two thirds of the anteroposterior length of the bulla (0); located at roughly one third of the anteroposterior length of the bulla (1).

178. Orientation of lateral furrow in lateral view: ventral (0); distinctly anteroventral (1).

179. Orientation of ventral keel of lateral lobe of bulla (*sensu* Boessenecker & Fordyce, 2015): faces ventrally (0); faces ventromedially or medially (1).

180. Anteroposterior outline of lateral lobe or main ridge of bulla: concave (0); straight or convex (1).

181 Position of involucral ridge in dorsal view: coincident with medial edge of the bulla (0); laterally retracted (1).

182. Sigmoid process deflected laterally in anterior or posterior view: absent (0); present (1); as state 1, but with the sigmoid process being nearly horizontal (2).

183. Dorsomedial corner of sigmoid process in anterior view: separated from the pedicle of the malleus (0); confluent with the pedicle of the malleus (1).

184. Ventral margin of sigmoid process in lateral view: present (0); absent, with the lateral margin of the sigmoid process turning smoothly into a sulcus on the lateral side of the bulla (1).

185. Shape of conical process in lateral view: well developed and dorsally convex (0); Reduced to a low ridge or absent (1).

186. Elliptical foramen: present (0); absent (1).

187. Medial lobe of tympanic bulla (*sensu* Boessenecker & Fordyce, 2015): present as distinct lobe and transversely wider than its lateral counterpart (0); present but subequal in width to the lateral lobe or smaller (1); absent or indistinct (2).

188. Crest connecting medial and lateral lobes of tympanic bulla in posterior view: present (0); absent (1).

189. Anteriormost point of involucral ridge: extends anteriorly to form the anteriormost point of the bulla (0); in line with or posterior to the anterior border of the bulla (1).

190. Dorsolateral surface of involucrum: divided into a low anterior and an elevated posterior portion, separated by a clearly defined step (0); forms a continuous rim (1).

191. Transverse creases on dorsal surface of involucrum: poorly developed or absent (0); well defined and deep (1).

192. Ridge on inside of bulla: extends laterally from involucrum and partially divides cavum tympani into anterior and posterior portions (0); absent (1).

193. Development of tympanic sulcus: developed as a faint line (0); forms a distinct crest or sulcus (1).

194. Outline and position of tympanic sulcus: forms a semicircular and ventrally curved line well separated from the intersection of the conical and sigmoid processes (0); forms a roughly horizontal line at or close to the level of the intersection of the conical and sigmoid processes (1).

195. Anteromedial portion of ventral surface of tympanic bulla: transversely convex (0); distinctly flattened or slightly concave (1).

196. Anterolateral corner of bulla: broadly rounded (0); inflated and forming a distinct lobe (1); flattened (2).

197. Anterolateral ridge or shelf: absent (0); present (1).

198. Position of posterior pedicle of tympanic bulla in dorsal view: situated at or near the posterior border of the bulla (0); located far anterior to the posterior end of the bulla (1).

**Mandible**

199. Posterior mandibular cheek teeth: oriented vertically or anteriorly (0); reclined posteriorly (1).

200. Medial surface of central part of mandible: similar to lateral surface (0); distinctly flattened relative to lateral surface (1).

201. Dorsomedial surface of posterior portion of mandibular body: flat or convex (0); distinctly excavated (1).

202. Mandibular symphysis: sutured or fused (0); unfused (1).

203. Outline of posterior portion of mandible in dorsal or ventral view: follows a straight line or simple curve (0); sigmoidal owing to a laterally reflexed neck and condyle (1).

204. Mandibular body in dorsal view: bowed medially (0); straight (1); bowed laterally, but with curvature mainly confined to anterior portion of mandible (2); evenly bowed laterally (3).

205. Anterior extremity of mandible: vertical or slightly twisted, with the ventral edge shifted medially (0); apex of mandible shifted to an almost horizontal position (1).

206. Mandibular body in medial or lateral view: height of ramus remains roughly constant throughout (0); arched dorsally (1); increases in height anteroposteriorly (2); dorsoventrally constricted near the centre (3); decreasing in height anteroposteriorly, with the anteriormost portion being distinctly expanded (4).

207. Height of mandibular foramen: dorsoventral height approximately that of the horizontal ramus, thus forming a mandibular fossa (0); dorsoventral height about half that of the horizontal ramus or less (1).

208. Anterior border of mandibular foramen: rounded (0); sharply triangular (1).

209. Dorsal border of mandibular foramen projected medially and developed into a roof: absent (0); present (1).

210. Satellite process (*sensu* Bisconti & Varola 2006): absent or limited to a low rugosity (0); present (1).

211. Relative position of anterior border of mandibular foramen: in line with coronoid process (0); posterior to coronoid process (1).

212. Subcondylar furrow (*sensu* Kimura, 2002): absent or extremely shallow (0); present as a well-defined groove medially only (1); as state 1, but with the dorsal border of the furrow being accentuated by a medially well-developed condyle (2); extends across the posterior surface of the condyle, separating it from the angular process both medially and laterally (3).

213. Coronoid process in lateral or medial view: forms a broad plate (0); distinctly triangular (1); shaped like a finger and pointing posteriorly (2).

214. Shape of coronoid process (if triangular) in lateral or medial view: sharply triangular and about as high dorsoventrally as long anteroposteriorly (0); bluntly triangular and considerably longer than high (1).

215. Anterior outline of coronoid process: vertical (0); bent laterally (1).

216. Postcoronoid elevation (*sensu* Struthers, 1889): absent (0); present (1).

217. Development of angular process in medial view: hollowed out (0); robust (1).

218. Anteroposterior position of angular process: located below the condyle, or slightly anterior (0); projects posteriorly beyond the condyle (1).

219. Angular process deflected ventrally: absent (0); present (1).

220. Fossa on medial side of angular process: absent (0); present (1).

221. Orientation of articular surface of mandibular condyle: posterior (0); posterodorsal (1); dorsal, with the condyle being confluent with a dorsoventrally expanded angular process (2); dorsal, with the condyle being larger than and clearly offset from the angular process (3).

222. Sulcus for attachment of mylohyoid muscle on ventromedial surface of mandible: absent (0); present (1).

**Vertebral column**

223. Height of transverse process of atlas at base: more than half the height of the articular surface (0); equal to half the height of the articular surface or less (1).

224. Foramen transversarium in axis: absent (0); present (1).

225. Development of parapophysis and diapophysis on axis in anterior or posterior view: parapophysis considerably more robust than diapophysis (0); parapophysis and diapophysis are similar in size (1).

226. Cervical vertebrae: separate (0); partially fused starting from the axis (1); completely fused (2).

227. Parapophysis on seventh cervical vertebra: present (0); absent (1).

228. Centra of cervical vertebrae in anterior or posterior view: rounded (0); squared (1).

229. Orientation of transverse processes of anterior lumbar vertebrae in anterior or posterior view: oriented distinctly ventrolaterally (0); oriented slightly ventrolaterally or subhorizontally (1); oriented laterally and horizontally (2).

230. Apices of neural spines of posterior thoracic and anterior lumbar vertebrae anteroposteriorly expanded and squared off: absent (0); present (1).

231. Number of lumbar vertebrae: more than 12 (0); 10 to 12 (1); fewer than 10 (2).

232. Metapophyses on posterior thoracic and anterior lumbar vertebrae in lateral view: oriented dorsally (0); oriented anterodorsally or anteriorly (1).

**Ribs and sternum**

233. Sternum: composed of several bones (0); composed of one bone (1).

234. Posterior ribs transversely expanded: absent (0); present (1).

**Forelimb**

235. Proportions of scapula: anteroposterior length of scapula approximately equals or is less than its maximum dorsoventral height (0); maximum anteroposterior length clearly exceeds its maximum dorsoventral height (1).

236. Coracoid process of scapula: present (0); absent (1).

237. Acromion process of scapula: present (0); absent (1).

238. Supraspinous fossa of scapula: present (0); absent or nearly absent, with acromion process located near anterior edge of scapula (1).

239. Deltoid crest of humerus: present as a distinct crest (0); absent or reduced to a variably developed rugosity (1).

240. Humerus: longer than or roughly the same length as radius and ulna (0); distinctly shorter than radius and ulna (1).

241. Orientation of humeral head in medial or lateral view: angled (0); vertical (1).

242. Distal portion of humerus in medial or lateral view: distal epiphysis narrower than shaft (0); distal epiphysis equal to or flared compared to shaft (1).

243. Articulation facet for radius on humerus in medial or lateral view: radial and ulnar facets are subequal in size (0); radial facet is distinctly larger than ulnar facet, excluding the olecranon (1).

244. Olecranon process: present as a distinct process (0); absent (1).

245. Manus: pentadactyl (0); tetradactyl (1).

**Hind limb**

246. Femur: present as a relatively well-developed bone (0); absent or reduced to a barely recognizable lump with an extremely rough surface texture (1).

247. Tibia: present (0); absent (1).

**Soft tissue**

248. Ventral throat grooves: absent (0); present and terminate well anterior to umbilicus (1); present and extend to umbilicus (2).

249. Ventral throat pouch: absent (0); present (1).

250. Tongue: muscular (0); reduced and predominantly connective tissue (1).

251. Temporomandibular joint: synovial (0); fibrocartilagenous mass originates in the glenoid fossa and envelopes the mandibular condyle (1).

252. Longitudinal ridges on rostrum: absent or indistinct (0); single median ridge (1); three longitudinal ridges (2).

253. Dorsal fin: present as fin or dorsal hump (0); absent (1).

254. Chromosome number: 42 (0); 44 (1).

255. Callosities: absent (0); present (1)

256. Coloration; dorsal blaze: absent (0); present (1)

257. Coloration ventral blaze: absent (0) present in <50% of the population (1) present in > 50% of the population (2)
