# Supplementary material for: A new member of fossil balaenid (Mysticeti, Cetacea) from the early Pliocene of Hokkaido, Japan
Source: R Soc Open Sci. 2020 Apr 22;7(4):192182. doi: 10.1098/rsos.192182 (PMC7211833; doi:10.1098/rsos.192182)
Supplement: Supplementary 4 [file rsos192182supp4.docx]

**Modified codings from Buono et al. (2017)**

This study modified codings on *“Balaenula” dosanko* (FCCP 1049) with direct examination. *“Balaenula” dosanko* was coded as “*Balaenua* sp.” in Buono et al. (2017).

**Skull**

13. Distinct pocket between the ascending process of the maxilla dorsally and the supraorbital process ventrally: absent (0); present (1).

*Archaeobalaena dosanko* 0 to ?

27. Teeth in adult individuals: present (0); absent or vestigial (1).

*Archaeobalaena dosanko* 1 to ?

31. Anterior edge of supraorbital process lateral to ascending process of maxilla with the skull in dorsal view: oriented transversely or pointing anteriorly (0); pointing posteriorly (1); linguiform and tapering to a point (2).

*Archaeobalaena dosanko* 0 to ? (not preserved)

33. Transverse width of anterior edge of supraorbital process lateral to ascending process of maxilla: longer than or equal to the combined transverse width of the adjacent rostral bones, as measured from the sagittal plane to the lateral border of the ascending process of the maxilla (0); shorter than the combined transverse width of the adjacent rostral bones (1).

*Archaeobalaena dosanko* 0 to ?

38. Postorbital process in dorsal view: oriented posteriorly (0); oriented laterally (1); oriented posterolaterally (2); short and not markedly projecting in any direction (3).

*Archaeobalaena dosanko* 1 to 0

*Balaenula astensis* 1 to 0

40. Orbital rim of supraorbital process of frontal in lateral view: dorsoventrally thin (0); thickened with a flat lateral surface (1); thickened with a rounded lateral surface (2).

*Archaeobalaena dosanko* 0 to 2

*Balaenula astensis* 0 to 2

73. Exposure of frontal on skull vertex: broadly exposed (0); anteroposteriorly compressed or absent (1).

*Archaeobalaena dosanko* - to 1

75. Outline of fronto-parietal suture: straight or lobate (0); frontals projects posteriorly along the sagittal plane and separate the left and right parietal anteriorly (1); highly irregular (2).

*Archaeobalaena dosanko* - to 1

91. Supramastoid crest of zygomatic process of squamosal (skull in lateral view): present (0); absent (1).

*Archaeobalaena dosanko* 1 to 0

**Periotic**

134. Anterior process of periotic in lateral view: squared off or rounded (0); triangular (1); anterior border of process is two-bladed and L-shaped (2).

*Archaeobalaena dosanko* 2 to 0

*Balaenula astensis* ? to 0

135. Shape of anteroventral angle of anterior process of periotic in medial or lateral view: rounded or forms a relatively blunt angle (0); slender and tapering to a point (1).

*Balaenula astensis* ? to 0

137. Dorsal deflection of anterodorsal corner of anterior process: absent (0); present (1).

*Archaeobalaena dosanko* 1 to ?

151. Promontorial groove on medial side of pars cochlearis: present, but relatively shallow (0); present and deeply excavated (1); present and forming a distinct constriction, separating a smooth and rounded ventral portion of the pars cochlearis from a flattened and striated dorsal one (2); absent (3).

*Archaeobalaena dosanko* 0 to 1

155. Anteroposterior alignment of proximal opening of facial canal, internal acoustic meatus and aperture for cochlear aqueduct: present (0); absent (1).

*Archaeobalaena dosanko* 1 to 0

170. Orientation of compound posterior process in ventral view, with periotic being in situ: oriented posterolaterally with respect to the longitudinal axis of the anterior process of the periotic (0); oriented at a right angle to the axis of the anterior process (1).

*Archaeobalaena dosanko* 1 to 0

**Tympanic bulla**

176. In situ orientation of main axes of tympanic bullae in ventral view: diverging posteriorly (0); parallel (1); diverging anteriorly (2).

*Archaeobalaena dosanko* - to 2

**Sternum**

233. Sternum: composed of several bones (0); composed of one bone (1).

*Archaeobalaena dosanko* 1 to ?
