# Supplementary figures and images for: A new member of fossil balaenid (Mysticeti, Cetacea) from the early Pliocene of Hokkaido, Japan
Source: R Soc Open Sci. 2020 Apr 22;7(4):192182. doi: 10.1098/rsos.192182 (PMC7211833; doi:10.1098/rsos.192182)

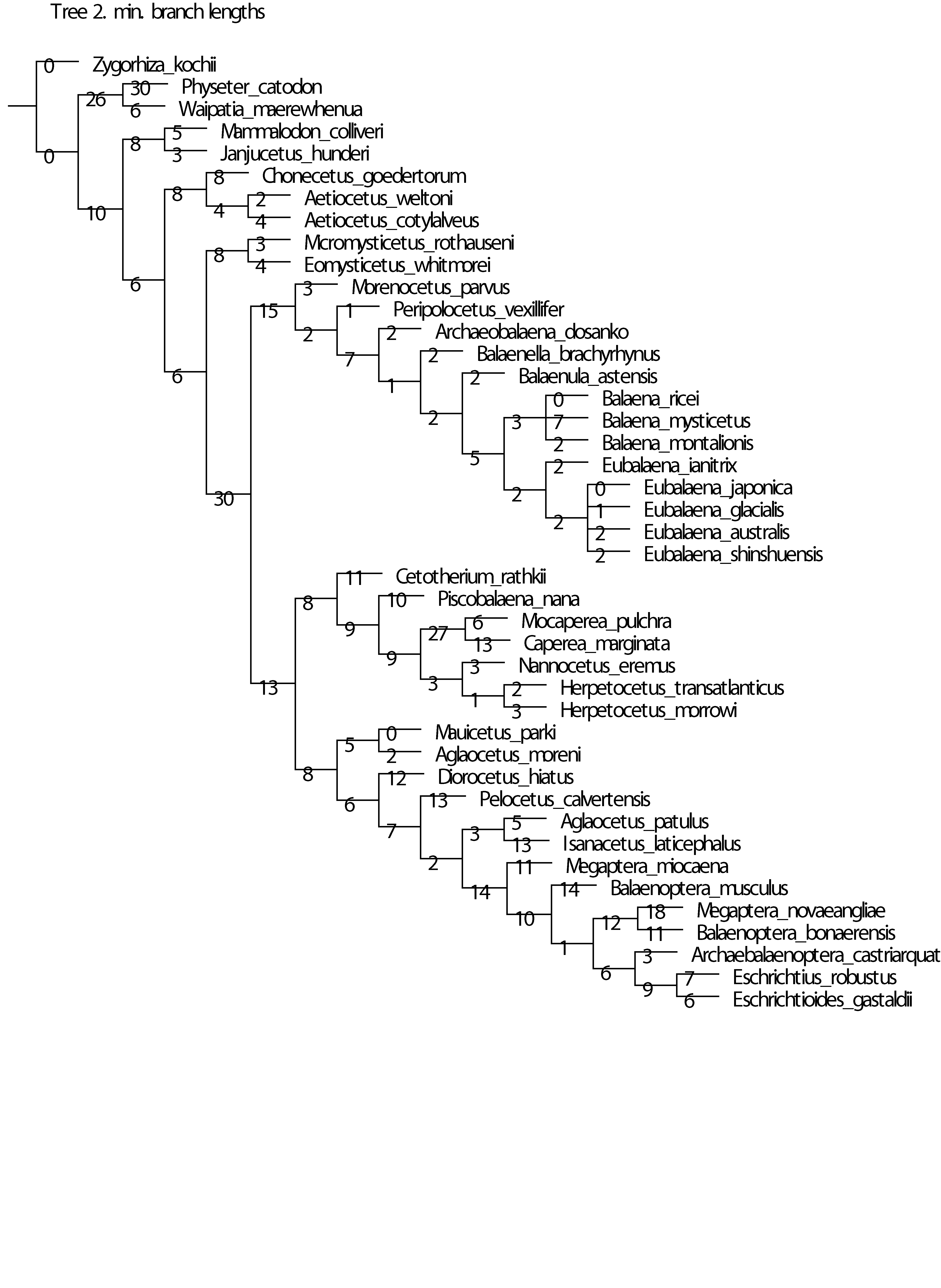

Supplement: Supplementary 5 [file rsos192182supp5.tif]
